# Heterochronic development of pelvic fins in zebrafish: possible involvement of temporal regulation of *pitx1* expression

**DOI:** 10.3389/fcell.2023.1170691

**Published:** 2023-08-24

**Authors:** Hilda Mardiana Pratiwi, Masahiro Hirasawa, Kohki Kato, Keijiro Munakata, Shogo Ueda, Yuuta Moriyama, Reiko Yu, Toru Kawanishi, Mikiko Tanaka

**Affiliations:** Department of Life Science and Technology, Tokyo Institute of Technology, Yokohama, Kanagawa, Japan

**Keywords:** fin development, zebrafish, heterochrony, poised enhancer, thyroid hormone

## Abstract

Anterior and posterior paired appendages of vertebrates are notable examples of heterochrony in the relative timing of their development. In teleosts, posterior paired appendages (pelvic fin buds) emerge much later than their anterior paired appendages (pectoral fin buds). Pelvic fin buds of zebrafish (*Danio rerio*) appear at 3 weeks post-fertilization (wpf) during the larva-to-juvenile transition (metamorphosis), whereas pectoral fin buds arise from the lateral plate mesoderm on the yolk surface at the embryonic stage. Here we explored the mechanism by which presumptive pelvic fin cells maintain their fate, which is determined at the embryonic stage, until the onset of metamorphosis. Expression analysis revealed that transcripts of *pitx1*, one of the key factors for the development of posterior paired appendages, became briefly detectable in the posterior lateral plate mesoderm at early embryonic stages. Further analysis indicated that the pelvic fin–specific *pitx1* enhancer was in the poised state at the larval stage and is activated at the juvenile stage. We discuss the implications of these findings for the heterochronic development of pelvic fin buds.

## Introduction

Heterochrony is defined as changes in the timing or rate of developmental processes (Alberch et al., 1979). Anterior and posterior paired appendages (forelimbs/pectoral fins and hindlimbs/pelvic fins) of vertebrates have been well studied as examples of heterochrony with respect to the relative timing of their development (Richardson et al., 2009). Forelimb buds develop earlier than hindlimb buds in most mammals, and this difference is amplified in marsupials, such as the brushtail possum (*Trichosurus vulpecula*), tammar wallaby (*Macropus eugenii*) and marsupial cat (*Dasyurus viverrinus*) (Bininda-Emonds et al., 2007; Richardson et al., 2009; Chew et al., 2014). In contrast, hindlimb buds develop earlier than forelimb buds in African clawed frogs (*Xenopus laevis*) and coqui frogs (*Eleutherodactylus coqui*) (Richardson et al., 2009). In teleost fishes, pelvic fin buds appear much later than pectoral fin buds. For instance, in zebrafish (*D. rerio*), pelvic fin buds emerge at 3 wpf during the larva-to-juvenile transition (metamorphosis), whereas pectoral fin buds arise from the lateral plate mesoderm on the yolk surface by 23 hpf (Kimmel et al., 1995; Grandel and Schulte-Merker, 1998).

Although pelvic fin buds of zebrafish appear at 3 wpf, fate mapping studies have shown that future pelvic fin bud cells are already determined by 16 hpf during early embryogenesis (Murata et al., 2010). The positional relationship between prospective pelvic fin cells on the yolk surface and the body trunk is shifted after trunk protrusion from the yolk sphere, at least in zebrafish, medaka and Nile tilapia (Murata et al., 2010; Kaneko et al., 2014), and such an allometric growth of the trunk may underlie the variation in pelvic fin position seen among teleost fishes (Tanaka, 2011). However, it remains uncertain how prospective pelvic fin cells maintain their fate for several weeks until the larva-to-juvenile transition.

In the hindlimb-forming region of chick and mouse embryos, *Pitx1*, which encodes a paired-like homeodomain protein, is expressed and plays critical roles in hindlimb skeletal morphogenesis (Lanctot et al., 1999; Szeto et al., 1999; Marcil et al., 2003; Duboc and Logan, 2011). Pitx1 acts in concert with the T-box transcription factor Tbx4 to control the outgrowth of hindlimb buds (Logan and Tabin, 1999; Duboc and Logan, 2011). In zebrafish and stickleback (*Gasterosteus aculeatus*), transcripts of *pitx1* and *tbx4* were detected specifically in pelvic fin buds during the larva-to-juvenile transition (Tamura et al., 1999; Cole et al., 2003; Don et al., 2016). Furthermore, stickleback that lack the pelvic fin–specific *pitx1* enhancer fail to form pelvic fins (Shapiro et al., 2004; Chan et al., 2010), and zebrafish deficient for *tbx4* function lack pelvic fins (Don et al., 2016; Lin et al., 2016), indicating the critical roles of both genes in the development of pelvic fins. Although the fate of the presumptive pelvic fin cells are determined during early embryogenesis (Murata et al., 2010), expression of neither *pitx1* nor *tbx4* has been reported in the presumptive pelvic fin field prior to the larva-to-juvenile transition period in any teleost fishes.

Pelvic fin buds emerge during the larva-to-juvenile transition (metamorphosis) in teleost fishes (Kimmel et al., 1995; Grandel and Schulte-Merker, 1998; Murata et al., 2010; Kaneko et al., 2014; Okamoto et al., 2018), and thyroid hormones (THs) are suggested to be the major factors promoting this transition among teleosts (Campinho, 2019). THs, such as thyroxine (T4) and triiodothyronine (T3), are secreted from the thyroid gland, and T4 can be converted to the more biologically active T3 (Yen et al., 2006; Koibuchi, 2009; Cheng et al., 2010). Treatment of larvae with exogenous T3 and/or T4 alters the juvenile pigmentation patterns in several teleosts (Parichy and Turner, 2003; McMenamin et al., 2014). In contrast, ablation of the thyroid partially blocks the juvenile transition in zebrafish (McMenamin et al., 2014). Based on these and other results, THs are proposed to play pivotal roles in triggering the larva-to-juvenile transition (Miwa et al., 1992; Power et al., 2001; Campinho, 2019); however, treatment of zebrafish larvae with THs alone has not been shown to accelerate the timing of initiation of pelvic fin development (Brown, 1997).

In this study, we explored the mechanisms by which presumptive pelvic fin cells maintain their cell fate, which is determined at early embryonic stages, until the larva-to-juvenile transition. We show that expression of *pitx1*, which plays pivotal roles in the initiation of the posterior paired appendages, briefly appears in the posterior lateral plate mesoderm including the presumptive pelvic fin cells on the yolk during the embryonic stage, but it soon became undetectable until the onset of metamorphosis. Further analysis indicated that the pelvic fin–specific *pitx1* enhancer is in a poised state at the larval stage and is activated at the juvenile stage. We discuss the implications of these findings for the heterochronic development of pelvic fin buds.

## Materials and methods

### Zebrafish embryos and larvae

Wild-type zebrafish (*Danio rerio*; strain TL) were reared and staged as described (Kimmel et al., 1995).

### Whole-mount *in situ* hybridization

Whole-mount *in situ* hybridization was performed as described (Westerfield, 2000). *D. rerio pitx1* (877 bp) and *tbx4* (653 bp) were amplified by PCR using the following primers, which hybridized to the indicated published sequence: *pitx1* (ENSDARG00000042785), 5′-GAC​GTC​TAT​CCC​ACC​TAC​AC-3′ and 5′-TGG​AAA​CAA​CAT​TAG​CAC​TG-3'; *tbx4* (NM_130914), 5′-TAT​TTG​CCT​CTG​AGA​CGA​AC-3′ and 5′-ACA​TAG​AGT​CTT​CCA​GGC​AT-3'.

### Hybridization chain reaction (HCR)

HCR probes were targeted *D. rerio pitx1* (NM_001040346) and *tal1* (NM_213237) and purchased commercially from Molecular Instruments, Inc. HCR assays were conducted according to (Ibarra-Garcia-Padilla et al., 2021). Images were obtained using an LSM 780 confocal microscope (Zeiss).

### Alignment of sequences

The sequence from the *pitx1* region of stickleback (*Gasterosteus aculeatus*) (BROAD S1 assembly), which includes a previously reported pelvic fin–specific enhancer sequence (Chan et al., 2010), was aligned against that of *D. rerio* (GRCz10 assembly), the medaka *Oryzias latipes* (MEDAKA1 assembly), the tetraodon *Tetraodon pustulatus* (TETRAODON 8.0 assembly), the cod *Gadus morhua* (gadMor1 assembly) and the fugu *Takifugu rubripes* (FUGU 4.0 assembly). These aligned sequences were visualized using mVISTA Genome Browser (http://genome.lbl.gov/vista/mvista/submit.shtml). Binding sites of transcription factors were predicted by MATCH (http://gene-regulation.com).

### Plasmid constructs

The zebrafish counterpart of the pelvic fin–specific *pitx1* enhancer (the *Pel* enhancer in Chan et al., 2010, later termed the *PelA* enhancer in Thompson et al., 2018) was amplified by PCR with the specific primers 5′-CTT​GAT​ATC​GAA​TTC​CTA​TGC​CTG​CTT​GTG​TCT​TTG-3′ and 5′- CGG​GCT​GCA​GGA​ATT​CCT​CTG​ATT​ATC​AAC​AGC​AGG-3′. The amplified fragment was then cloned into the EcoRI sites of the pBSSK–vector (pBSSK-*DrPel*) via the In-Fusion reaction (Clontech). pT2AL200R150G (Urasaki et al., 2006) was digested with *Sal*I, blunt-ended using T4 DNA polymerase and ligated (pT2AL200R150G-without *Sal*I). Then, the basal promoter of EF1α was amplified with specific primes 5′-CCC​AAG​CTT​CTA​GAA​CTC​GCC​GCA​GAC​CC-3′ and 5′-CCG​CTC​GAG​ACG​CGT​CGA​CCA​TGC​AAG​CTA​GCT​TAT​GAC​GC-3′ using pT2AL200R150G as the template. This amplified fragment was then cloned into the *Xho*I and *Hind*III sites of pT2AL200R150G-without *Sal*I (pT2AL-*EF1α*min-*EGFP*). pBSSK-*DrPel* was digested with *Not*I and blunt-ended using T4 DNA polymerase and then was digested with *Xho*I and cloned into the *Sma*I site of pT2AL-*EF1α*min-*EGFP* (pT2AL-*DrPel-EF1α*min-*EGFP*). Then, pBSSK-*DrPel* was digested with *EcoR*I and *Sma*I and cloned into the *Sma*I site of pT2AL-*DrPel-EF1α*min-*EGF* (pT2AL-*DrPel* × 2*-EF1α*min-*EGFP*).

### Microinjection

For enhancer analysis, the plasmids of interest were co-injected with mRNA that encodes *Tol2* transposase (Kawakami, 2004) into one-cell-stage embryos. Injected embryos were raised to adulthood and screened for germline transmission. Founders that expressed enhanced green fluorescent protein (EGFP) were crossed with TL fish, and the resulting embryos were screened for EGFP expression in the pelvic fin.

### Chromatin immunoprecipitation and quantitative real-time PCR (ChIP-qPCR)

ChIP was performed as described (Visel et al., 2009; Nakato et al., 2013; Suda et al., 2014) with the following modifications. The presumptive pelvic fin field from ∼200 larvae at 2 wpf [total length (TL), 5.5–7.0 mm; Supplementary Figure S1A] and the early pelvic fin from ∼100 juveniles at 3–4 wpf (TL, 9.0–12.0 mm; Supplementary Figure S1B) were dissected (green areas shown in Figure 2C). These tissues were fixed in 1% formaldehyde (for H3K27ac and H3K27me3) or in 1% formaldehyde with 2 mM *N*,*N*′-disuccinimidyl glutarate (for CBP) for 10 min at room temperature, washed with phosphate-buffered saline (PBS) and stored at −80°C. ChIP was performed using these tissue samples with antibodies against H3K27ac (#ab4729, Abcam), H3K27me3 (#ab39536, Abcam) or CBP (GTX101249, GeneTex). Samples were homogenized in lysis buffer 1 [20 mM Tris-HCl (pH 7.5), 10 mM NaCl, 2.5 mM MgCl_2_, 0.2% nonyl phenoxypolyethoxylethanol 40, 1% protease inhibitor, 1% phenylmethanesulfonyl fluoride (PMSF)]. After centrifugation, pellets were suspended in the zebrafish lysis buffer [50 mM Tris-HCl (pH 8.0), 10 mM EDTA, 1% SDS, 1% protease inhibitor, 1% PMSF]. Samples from the whole-cell extract and ChIP fractions were sheared with an ultra sonicator (10 × 10 s and 30% amplitude; BRANSON SONIFIRE 250D). Sequences were amplified with the following primers: *pitx1* enhancer (Chr21: 47,921,810–47,921,997), 5′-TTG​TCA​CAC​CTT​TAC​TTC​GGA-3′ and 5′-AAC​GGA​GAG​CGT​CTG​TTG​G-3′; positive control, β-actin (*actb*) promoter (Chr3: 40,386,829–40,386,986) (Lindeman et al., 2010), 5′-CAG​TCG​GTT​CAG​TTC​ATA​GT-3′ and 5′-GGT​TGT​GTA​ATT​GAT​CGC​AG-3′; negative control, open reading frame (ORF)-free *pitx1* enhancer intergenic region (IGR) (Chr21: 45,736,667–45,736,860), 5′-GTG​GGT​GGT​GAT​TTC​GTG​TC-3′ and 5′- GCC​TTT​GTT​GCT​CTC​TGT​CA-3′. The mean ± standard deviation (s.d.) was calculated, and a statistical analysis was performed using Student’s *t*-test.

### Treatment with hormones

For making stock solution, 10 mg of l-thyroxine (T4; SIGMA), or 20 mg of 3,3′,5-triiodo-l-thyronine (T3; SIGMA) was dissolved in 200 μL of 2N NaOH and added 800 μL of water, 10 mg of hydrocortisone (HC; Wako) was dissolved in 1 mL of ethanol and added 9 mL of water, and 1 mg of 9-*cis*-retinoic acid (RA; Funakoshi) was dissolved in 2 mL of ethanol. Each solution was diluted in water to the final administrated concentrations. The same amount of diluted NaOH solution was added to the control. Final administrated concentrations are as follows: T4 100 μM; T3 118 μM; RA 0.5 μM; HC 250 μM. For each condition, 10 larvae at 2 wpf (TL, 5.5–7.0 mm) were kept in 40 mL of fish water.

## Results

### Expression of *pitx1* in developing zebrafish embryos, larvae and juveniles

**FIGURE 1:**
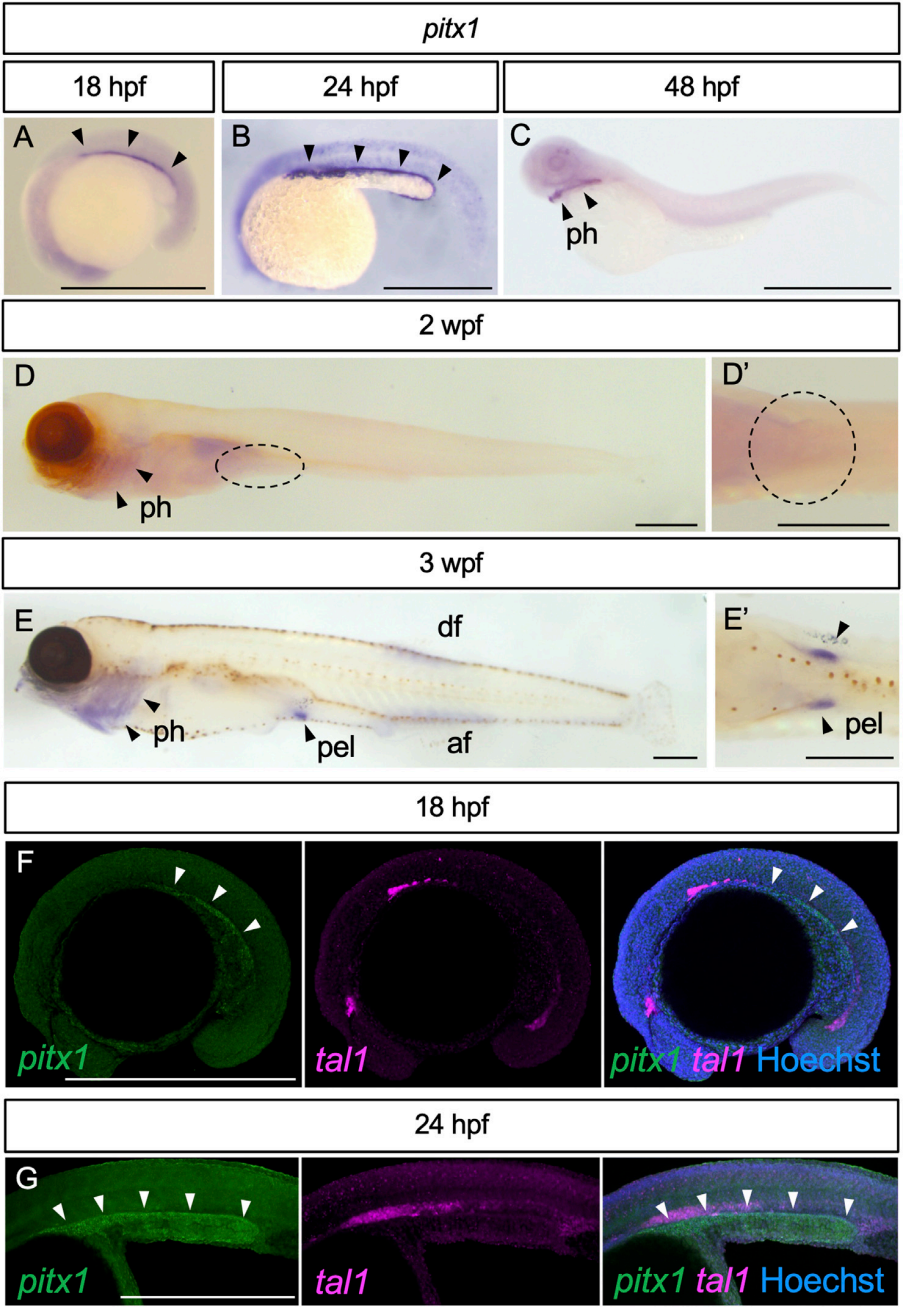
Expression of *pitx1* in zebrafish. **(A–E)** Representative images of *pitx1* expression in zebrafish at 18 hpf **(A)** (*n* = 3), 24 hpf **(B)** (*n* = 4), 48 hpf **(C)** (*n* = 3), 2 wpf **(D)** (*n* = 4) and 3 wpf **(E)** (*n* = 3). **(D′,E′)** Ventral views of the pelvic fin region in **(D,E)**, respectively. **(F,G)** HCR *in situ* images of 18 hpf **(F)** and 24 hpf **(G)** embryos with *pitx1* and *tal1* signals. af, anal fin; df, dorsal fin; pel, pelvic fin; ph, pharynx. Scale bars, 0.5 mm.

We first examined the expression pattern of *pitx1*, known to be involved in the initiation of pelvic fin development in stickleback (Shapiro et al., 2004; Chan et al., 2010), in developing zebrafish embryos, larvae and juveniles (Figures 1A–E). At 18 hpf, *pitx1* expression appeared in the posterior lateral plate mesoderm (arrowheads in Figure 1A). By 24 hpf, expression of *pitx1* was intensified in the posterior lateral plate mesoderm (arrowheads in Figure 1B). By 48 hpf, *pitx1* expression was hardly detectable in the lateral plate mesoderm, whereas it was detected in the pharyngeal region (arrowheads in Figure 1C), as in other teleosts (Shapiro et al., 2004; Tanaka et al., 2005; Chan et al., 2010). At 2 wpf (TL, 5.5–7.0 mm; Supplementary Figure S1A), *pitx1* transcripts were still detectable in the pharyngeal region (arrowheads in Figure 1D) but not in the presumptive pelvic fin field (dashed oval in Figures 1D, 1D′). By 3 wpf (TL, 9.0–12.0 mm; Supplementary Figure S1B), expression of *pitx1* was detected in the pelvic fin bud, as well as in the pharyngeal region, dorsal fin and anal fin (Figures 1E, 1E′).

Detection of *pitx1* expression in the posterior lateral plate mesoderm during early embryogenesis is interesting. However, we cannot exclude the possibility that *pitx1*-positive cells are hemanigioblastic cells (precursors of blood and endothelial cells) derived from the posterior lateral plate mesoderm (Xiong, 2008). To address this possibility, we compared the expression of *pitx1* with that of hemangioblast marker *tal1* (also known as *scl*) (Gering et al., 1998) in early embryos by using Hybridization Chain Reaction (HCR) probe sets (Figures 1F, G). In early embryos, *pitx1* signals were detected in the posterior lateral plate mesoderm, and not overlapped with *tal1* signals (Figures 1F, G), suggesting that these *pitx1*-positive cells are at least not hemangioblastic cells.

Our results suggest that, in zebrafish, expression of *pitx1* is activated in the posterior lateral plate mesoderm during early embryonic stages, is repressed during larval stages and re-appears in the pelvic fin bud during juvenile stages.

### Pelvic fin–specific *pitx1* enhancer is “poised” until the larva-to-juvenile transition

**FIGURE 2:**
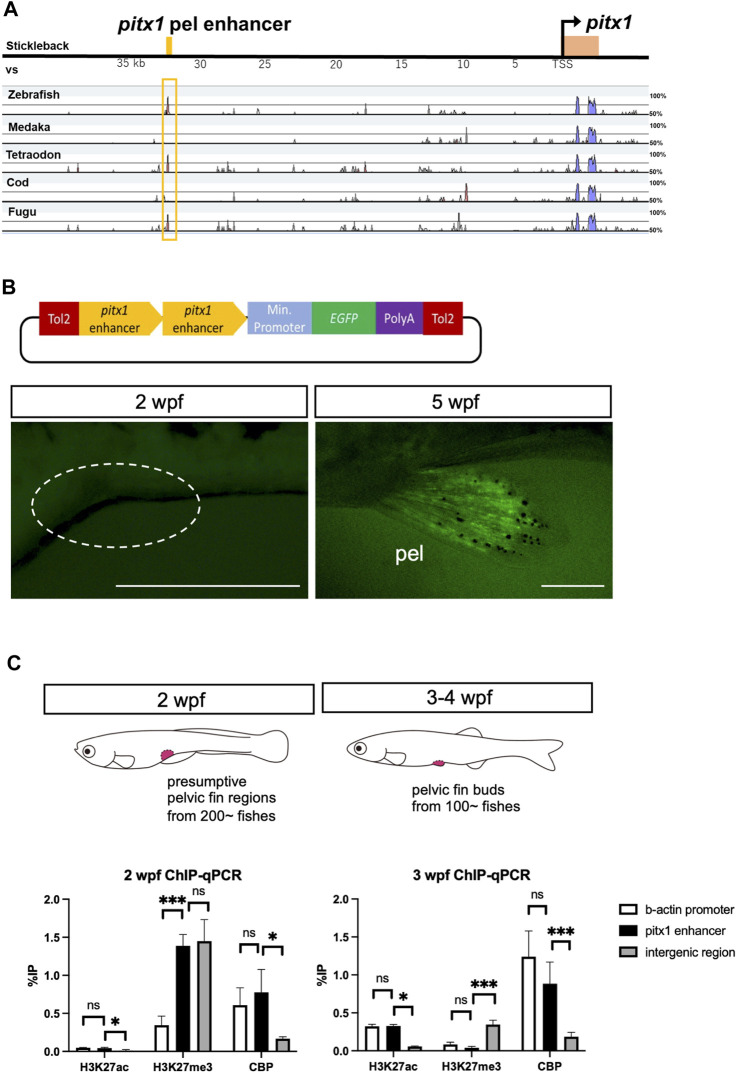
Pelvic fin–specific *pitx1* enhancer is in a poised state until metamorphosis. **(A)** mVISTA alignment of sequences from the *pitx1* region from stickleback, medaka, tetraodon, cod and fugu. Red peaks indicate >50% sequence identity in a 50-bp sliding window. The yellow bar at the top indicates the 501-bp pelvic fin–specific enhancer region identified 35 kb upstream of *pitx1* in stickleback (*Pel-*501 bp in Chan et al., 2010). **(B)** *DrPel* enhancer does not drive *EGFP* expression in 2-wpf zebrafish larvae, whereas it does drive *EGFP* expression specifically in the pelvic fins of 5-wpf juveniles. Scale bars, 0.5 mm. **(C)** Epigenetic states of *DrPel* at different developmental time points were revealed by ChIP-qPCR analysis of the presumptive pelvic fin region from 2-wpf larvae and the pelvic fin bud from 3- to 4-wpf juveniles. The *β-actin* promoter sequence and the ORF-free IGR were used as positive and negative control regions, respectively. *p < 0.05, ***p < 0.001; ns, not significant. Mean ± s.d. (*n* = 3). See text for more details.

To investigate how the expression of *pitx1* was repressed during larval stages, we assessed the function of the enhancer of *pitx1* in driving its expression in the pelvic fin bud. For this, we compared the sequence from the *pitx1* region in stickleback, including the pelvic fin–specific enhancer sequence (*Pel* enhancer in Chan et al., 2010), with that from zebrafish, medaka, tetraodon, cod and fugu (Figure 2A). This allowed us to identify the homolog of the pelvic fin–specific *pitx1* enhancer in zebrafish ∼35 kilobase pairs (kbp) upstream of *pitx1* (Figure 2A), which contains the predicted multiple transcription factor binding sites (Supplementary Figure S2). To assess the function of this putative enhancer, we cloned the 837-bp fragment in front of a basal promoter followed by an *EGFP* reporter gene (Figure 2B). This fragment did not drive *EGFP* expression in 2-wpf larvae (0/17 in F2 fishes; Figure 2B), whereas it did drive *EGFP* expression specifically in the pelvic fin in 4- to 5-wpf juveniles (5/17 in F2 fishes; Figure 2B). LPM-specific EGFP expression was not detected in 24 hpf-F0 founders or F1 fish (data not shown). These results suggest that the identified 837-bp non-coding sequence 35 kbp upstream of *pitx1* contains a specific enhancer for pelvic fin expression in zebrafish, which we term the *DrPel* enhancer.

Poised enhancers have been suggested to be involved in the future activation of developmental genes in cells upon their differentiation into a specific cell fate (Rada-Iglesias et al., 2011; Crispatzu et al., 2021). In human embryonic stem cells, poised enhancers are enriched with universal co-activators, such as p300 (Kamei et al., 1996) and CBP, and display high chromatin accessibility; however, unlike active enhancers, they are not marked with acetylation of histone H3 at lysine 27 (H3K27ac) but are enriched for trimethylation of histone H3 at lysine 27 (H3K27me3), which is associated with Polycomb group protein complexes (Rada-Iglesias et al., 2011). Such poised enhancers have also been identified in chicken and mouse embryos (Crispatzu et al., 2021). Thus, we explored the possibility that the *DrPel* enhancer of presumptive pelvic fin cells of zebrafish larvae is already in a poised state prior to the emergence of the pelvic fin buds. We dissected the presumptive pelvic fin region from ∼200 larvae at 2 wpf (TL, 5.5–7.0 mm) and the pelvic fin bud from ∼100 juveniles at 3–4 wpf (TL, 9.0–12.0 mm) and performed ChIP with H3K27ac, H3K27me3 or CBP antibodies. Subsequently, qPCR was performed with a pair of primers that amplified a region within the *DrPel* enhancer, as well as two pairs of primers that amplified the *β-actin* promoter (Lindeman et al., 2010) or the ORF-free IGR (see details in *Materials and Methods*) as control regions. ChIP-qPCR results demonstrated the absence of H3K27ac and the enrichment of H3K27me3 in the *DrPel* sequence of the presumptive pelvic fin region of 2-wpf larvae (Figure 2C). Whereas we observed a large increase in H3K27ac levels in the *DrPel* sequence in the pelvic fin bud of juveniles at 3–4 wpf, H3K27me3 levels were hardly detectable (Figure 2C). We then examined the association of a universal transcriptional co-activator, CBP, with the *DrPel* enhancer to evaluate whether the enhancer was silenced, poised or activated (Rada-Iglesias et al., 2011). Our results showed that both in the presumptive pelvic fin region at 2 wpf and in the pelvic fin bud at 3–4 wpf, the *DrPel* enhancer was associated with high levels of CBP (Figure 2C), suggesting that the *DrPel* enhancer in the presumptive pelvic fin is in a poised state at 2 wpf and is activated in the pelvic fin bud at 3–4 wpf.

### Exogenous thyroid hormones are not sufficient for the induction of *pitx1* in the presumptive pelvic fin

**FIGURE 3:**
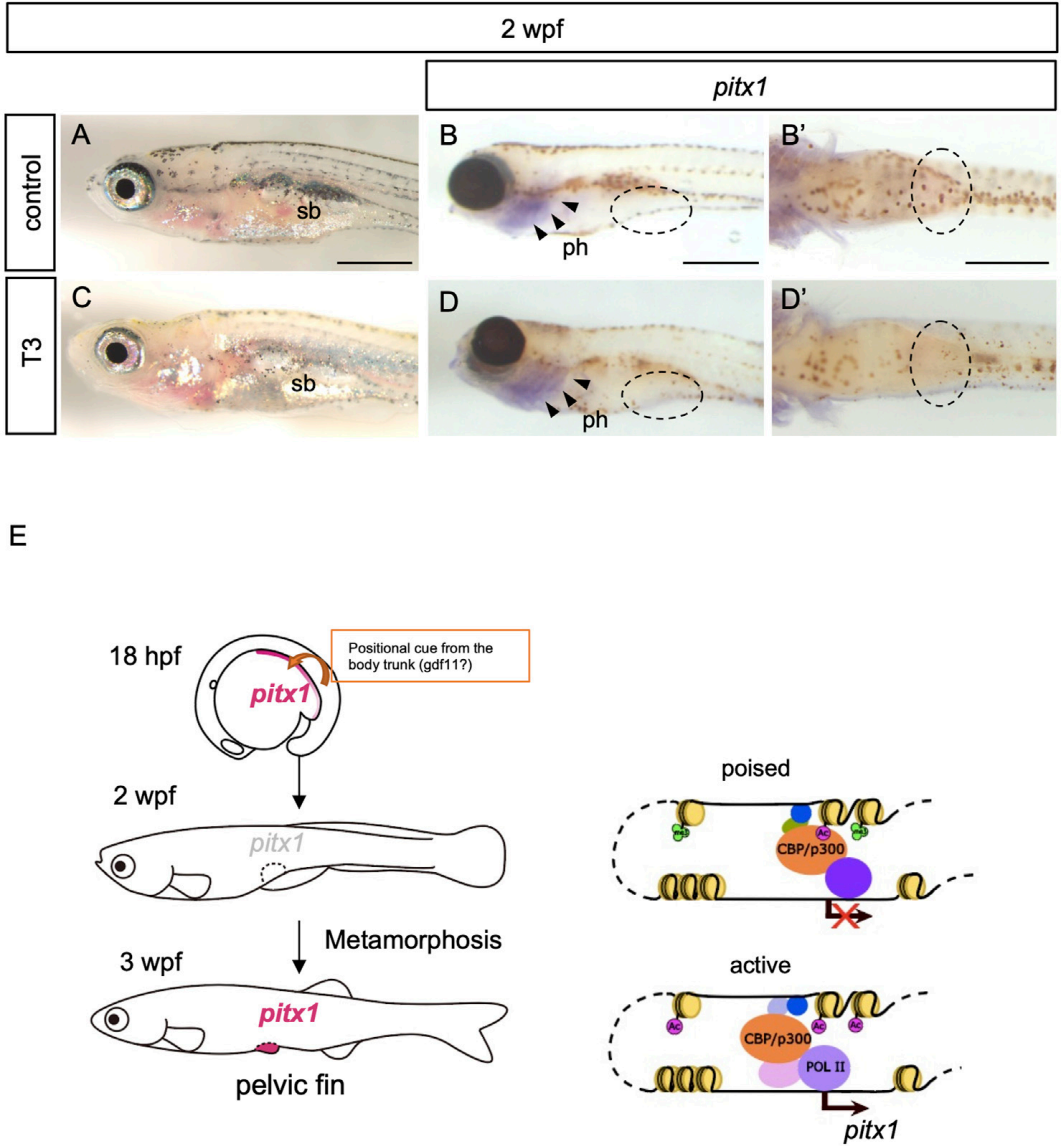
Exogenous thyroid hormones are not sufficient for the induction of *pitx1* in the presumptive pelvic fin. **(A–D)** Zebrafish larvae at 2 wpf were treated with active T3 **(C,D)** or with vehicle (control; **(A,B)** for 24 h. Both the resulting pigment pattern [**(A)**, *n* = 4; **(C)**, *n* = 3] and the expression of *pitx1* [**(B)**, *n* = 4; **(D)**, *n* = 3] were examined. **(B′,D′)** Ventral views indicated by the dashed ovals in **(B,D)**, respectively. Expression of *pitx1* was observed in the pharyngeal region [arrowheads in **(B,D)**] but not in the presumptive pelvic fin region of either T3-treated or control larvae [dashed ovals in **(B,D)**]. ph, pharynx; sb, swim bladder. Scale bars, 0.5 mm. **(E)** A proposed model of how *pitx1* expression is controlled in the presumptive pelvic fin regions. Presumptive pelvic fin cells are likely to receive a positional cue, probably gdf11 or an unknown factor controlled by gdf11, from the body trunk by at least 16 hpf (Murata et al., 2010; Tanaka, 2011). *pitx1* expression appears in the lateral plate mesoderm, including the presumptive pelvic fin cells, at least by 18 hpf. However, at 2 wpf, the *DrPel* enhancer becomes in a poised state, and thus *pitx1* expression is not activated within the presumptive pelvic fin region. The *DrPel* enhancer is activated in the pelvic fin buds during the larva-to-juvenile transition (metamorphosis).

Development of the pelvic fin bud initiates at 3 wpf during the larva-to-juvenile transition (metamorphosis) in zebrafish (Grandel and Schulte-Merker, 1998). Although an application of T3 or T4 alone does not accelerate the development of pelvic fin buds in zebrafish (Brown, 1997), thyroid hormones have been shown to play pivotal roles in metamorphosis in all teleost fishes studied (Campinho, 2019). We thus considered whether thyroid hormones are involved in the activation of *pitx1* transcription in the pelvic fin bud during metamorphosis. To test this hypothesis, we incubated 2-wpf zebrafish larvae with 100 μM T4 (the biologically less-active thyroid hormone), with 100 μM T3 (active thyroid hormone) or with T3 with endocrine disruptors that mimic thyroid hormone activity, such as 0.5 μM 9-*cis*-RA and/or 250 μM hydrocortisone (HC) and examined the expression of *pitx1* (Figures 3A–D; Supplementary Figure S3). After 24 h of incubation, decreased melanophore pigmentation was observed in the skins of 2-wpf larvae treated with T3 or with T3 and RA or HC, but not in those of control or T4-treated larvae (Figures 3A, C; Supplementary Figure S3). This suggests that these treatment conditions are sufficient to affect larval pigment development, as seen in several teleosts (Yoo et al., 2000; Parichy and Turner, 2003; McMenamin et al., 2014; Salis et al., 2021). Thus, we incubated 2-wpf larvae with the same concentrations of hormones and endocrine disruptors for 24 h and examined the expression pattern of *pitx1* in the larvae. Although *pitx1* expression was observed in the pharyngeal regions of 2-wpf larvae incubated with T3 or with T3 and RA or HC, as was observed in control larvae, no *pitx1* transcripts were detected in the presumptive pelvic fin region in any of these larvae (Figures 3B, D; Supplementary Figures S3A–J). These results suggest that none of these hormones or endocrine disruptors are sufficient for the induction of *pitx1* expression in the pelvic fin region of 2-wpf larvae, at least under these conditions.

## Discussion

Our data indicate that, in zebrafish, expression of *pitx1* appears in the posterior lateral plate mesoderm during the embryonic stage, but becomes downregulated during the larval stage. We further showed that the pelvic fin–specific *pitx1* (*DrPel*) enhancer is in a poised state in presumptive pelvic fin cells of larvae, and becomes activated in pelvic fin buds of juveniles during the metamorphosis (Figure 3E).

In zebrafish, pectoral fin buds appear as bulges of mesenchymal cells on the yolk surface at around 23 hpf, whereas pelvic fin buds emerge in the ventral body wall at 3 wpf, during the larva-to-juvenile transition (Kimmel et al., 1995; Grandel and Schulte-Merker, 1998). However, the fate of the presumptive pelvic fin cells has already been determined at least by 16 hpf and is maintained for 3 weeks by an unknown mechanism (Murata et al., 2010). In tetrapod embryos, the caudal expression of *Growth differentiation factor 11* (*Gdf11*) is critical for the positioning of hindlimb-forming fields along the body axis (McPherron et al., 1999; Liu, 2006; Jurberg et al., 2013; Matsubara et al., 2017). In zebrafish, expression of *gdf11* appears in the caudal end of the body trunk during early embryogenesis (10 hpf), and a reduction in *gdf11* transcripts results in a caudal displacement of the pelvic fin position (Murata et al., 2010), suggesting that the function of *gdf11* for defining the position of posterior paired appendages is conserved in zebrafish, even though the expression of *gdf11* persists only during early embryogenesis. In this study, we showed that the *pitx1* expression in the posterior region of the lateral plate mesoderm also persisted during early embryogenesis. The *pitx1*-positive region observed at 18 hpf (Figure 1A) is consistent with the presumptive pelvic fin cells as determined by fate-mapping experiments (Murata et al., 2010). Therefore, it is likely that the presumptive pelvic fin cells are already determined by *pitx1* at the early embryonic stage, but its expression is not sufficient for the induction of pelvic fin buds. Indeed, *tbx4*, another factor required for the development of hindlimbs (Logan and Tabin, 1999; Duboc and Logan, 2011), was not detected in the lateral plate mesoderm of early zebrafish embryos (Supplementary Figure S4), as reported (Tamura et al., 1999; Ruvinsky et al., 2000). The inability of *tbx4* induction during early embryogenesis may explain the heterochronic development of pelvic fins. In zebrafish, the *pitx1*-positive-posterior region of the lateral plate mesoderm apparently does not split into the somatic and splanchnic layers at the embryonic stage. Subdivision of the lateral plate mesoderm into the somatic and splanchnic layers is suggested to be the critical step for limb bud formation (Onimaru et al., 2011; Tanaka and Onimaru, 2012; Tanaka, 2013). Therefore, the unseparated lateral plate mesoderm at the posterior region of the embryo might also be one of the reasons why the *pitx1*-positive cells do not initiate the development of pelvic fin buds during embryogenesis in zebrafish.

The results presented here demonstrate that the *DrPel* enhancer is in a poised state in the presumptive pelvic fin cells of larvae, and activated during the metamorphosis. Although it is tempting to speculate that the poised state of the *DrPel* enhancer is related to the maintenance of the fate of pelvic fin cells, it is difficult to ascertain due to the limited knowledge of the regulatory mechanism of metamorphic development in teleosts. Metamorphosis is shown to be triggered by thyroid hormones in all teleost fishes studied (Campinho, 2019). In amphibians, the presence of thyroid hormones induces the removal of HDAC-containing corepressor complexes from thyroid hormone receptors (TRs) (Chen and Evans, 1995; Horlein et al., 1995), and the recruitment of histone acetyltransferase (HAT; renamed as lysine acetyltransferase, KAT) coactivators such as p300 and CBP for activation of the transcription of target genes (Glass and Rosenfeld, 2000). This cofactor exchange identified during the metamorphosis of *Xenopus laevis* has been suggested to control the timing of metamorphic development of various organs (Sachs et al., 2002; Tomita et al., 2004). Thus, it is interesting to speculate that the heterochronic development of pelvic fins during the metamorphosis of zebrafish is regulated by the similar mechanisms, however, the application of thyroid hormones is not sufficient for accelerating the induction of pelvic fin formation (Brown, 1997), or *pitx1* expression (Figure 3) in zebrafish larvae. In our study, the *DrPel* enhancer in presumptive pelvic fin cells of 2-wpf larvae had already recruited CBP, and thyroid hormones are not sufficient for the activation of *pitx1* expression in the pelvic fin cells. Although we cannot exclude the possibility that TRs are directly bound to the *DrPe*l enhancer, thyroid hormone response element (TRE) sequences were not identified within *DrPel*, at least in our survey, while a predicted retinoid X receptor (RXR) binding site was found (Supplementary Figure S2). It is still possible that another enhancer or promoter topologically associated with the *DrPel* is silenced by unliganded TRs, or that TRs are involved in regulating the transcription of unknown factor(s) directly or indirectly controlling the chromatin state of the *DrPel* enhancer. Future studies are needed to identify unknown factor(s) required for *pitx1* induction and to investigate the temporal association of chromatin structural modifications of the pelvic fin/posterior paired appendages–specific *pitx1* enhancer. The delayed timing of pelvic fin development in teleosts was suggested to be correlated with variation in the pelvic fin position seen among teleost fishes, which ranges from the jugular to abdominal level (Murata et al., 2010; Tanaka, 2011; Kaneko et al., 2014). Such a variation in the pelvic fin position is a notable feature of teleost species from behavioral and taxonomic viewpoints (Gosline, 1971; Rosen, 1982; Standen, 2008). Elucidating the regulatory mechanism of the pelvic fin–specific *pitx1* enhancer is expected to provide insights into the diversification of pelvic fin position among teleosts.

## Data Availability

The original contributions presented in the study are included in the article/Supplementary Material, further inquiries can be directed to the corresponding author.
