# Supplementary material for: Heterochronic development of pelvic fins in zebrafish: possible involvement of temporal regulation of pitx1 expression
Source: Front Cell Dev Biol. 2023 Aug 24;11:1170691. doi: 10.3389/fcell.2023.1170691 (PMC10483283; doi:10.3389/fcell.2023.1170691)
Supplement: Supplementary file 1 [file DataSheet1.PDF]

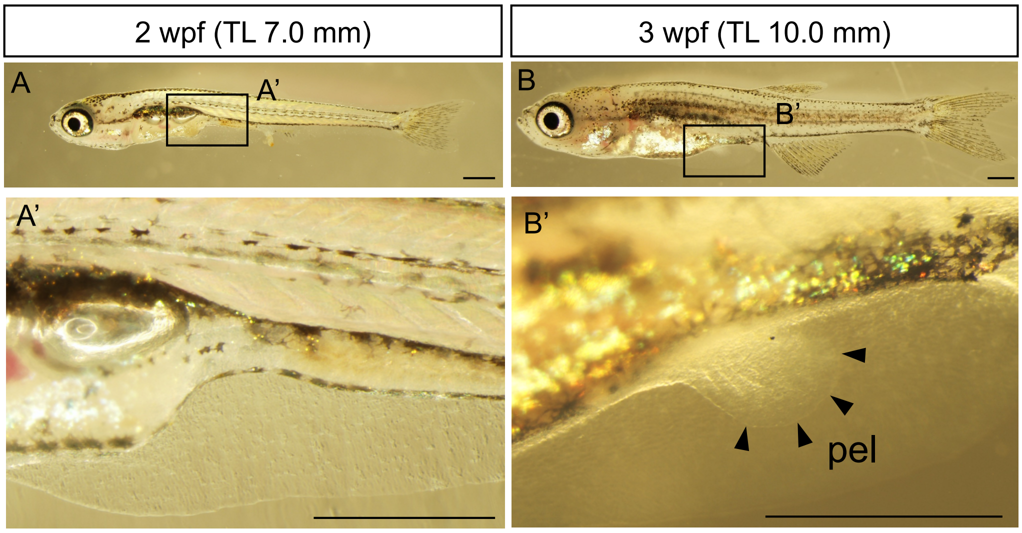

Supplementary Figure 1. Zebrafish larva and juvenile. (A, B) Lateral view of a larva at 2 wpf at 7.0 mm TL (A) and of a juvenile at 3 wpf at 10.0 mm TL (B). (A’, B’) Magnified images in (A) and (B), respectively. pel, pelvic fin. Scale bars, 0.5 mm.

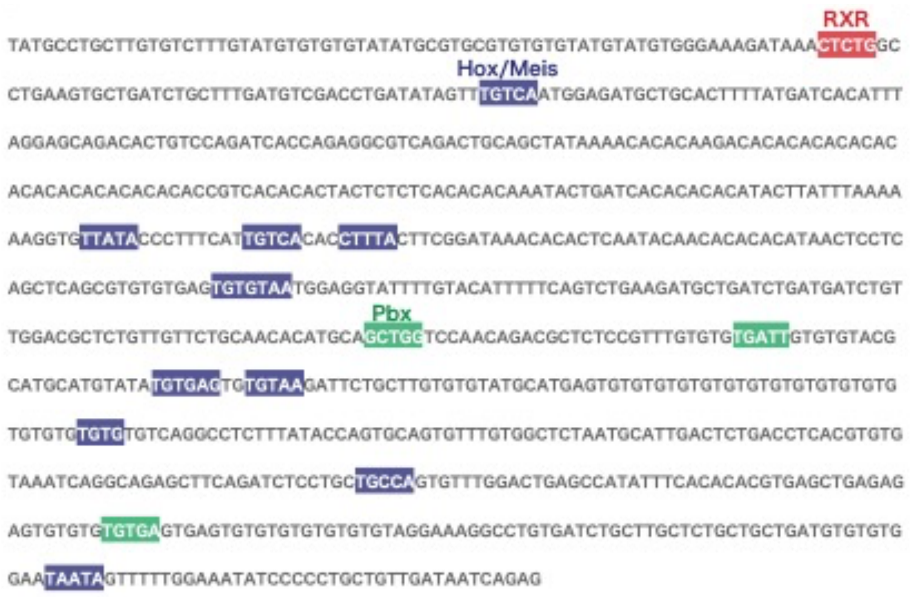

Supplementary Figure 2. Predicted binding sites of transcription factors within the pelvic fin–specific *pitx1* enhancer (873 bp). Binding sites of transcription factors were predicted by MATCH.

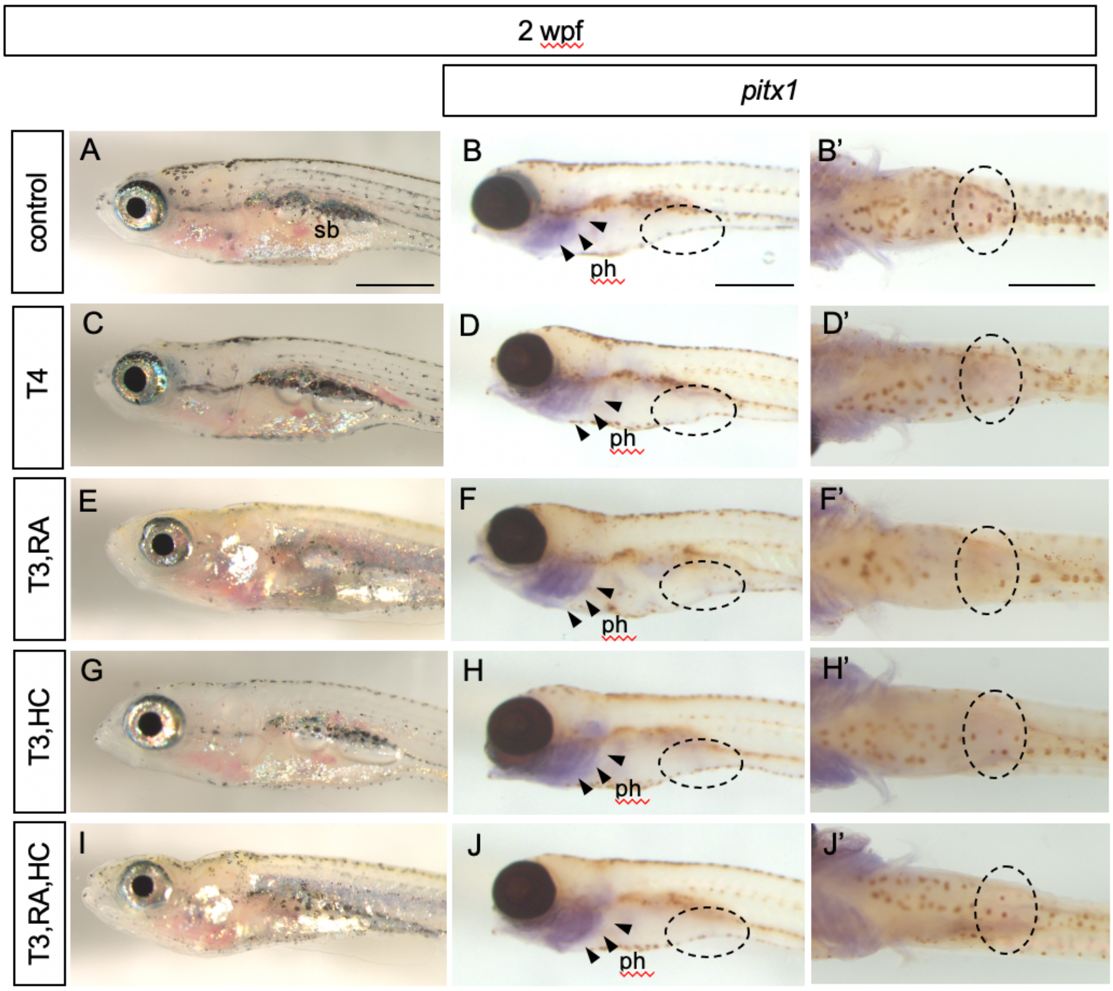

Supplementary Figure 3. Analysis of zebrafish larvae treated with hormones. (A-J) Zebrafish larvae at 2 wpf were treated with inactive T4 (C, D), active T3 and retinoic acid (RA) (E, F), active T3 and hydrocortisone (HC) (G, H), active T3, RA and HC (I, J) or with vehicle (control; A, B) for 24 h. The pigment pattern (A, n=4; C, n=5; E, n=3; G, n=3; I, n=3) and expression of *pitx1* (B, n=4; D, n=5; F, n=3; H, n=3; J, n=3) were then examined. (B', D', F', H', J') Ventral views indicated by the dashed ovals in (B), (D), (F), (H) and (J), respectively. Expression of *pitx1* was observed in the pharyngeal region (arrowheads in B, D, F, H, J) but not in the presumptive pelvic fin region under any treatments (dashed ovals in B, D, F, H, J). The control figures (A, B) are the same as Figure 3A, B. ph, pharynx; sb, swim bladder. Scale bars, 0.5 mm.

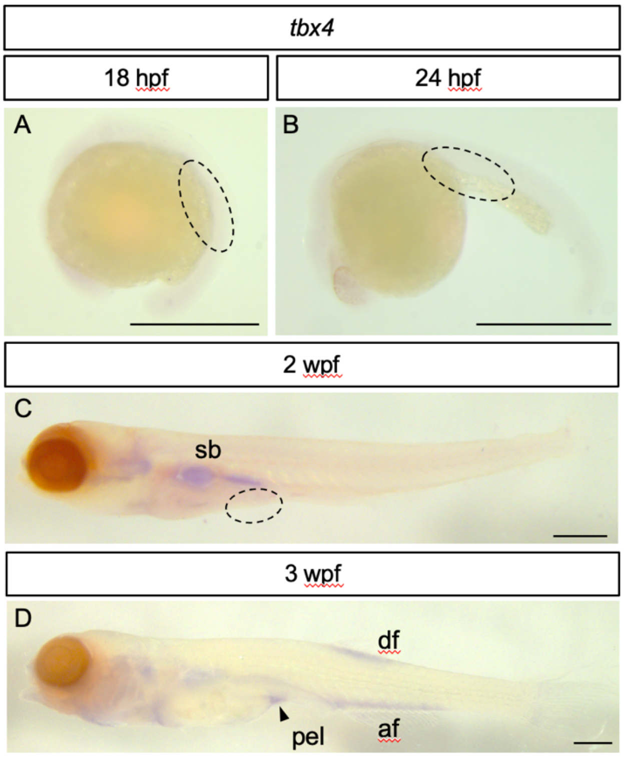

Supplementary Figure 4. Expression of *tbx4* in zebrafish. **(A-D)** Representative images of *tbx4* expression in zebrafish at 18 hpf **(A)** (n = 3), 24 hpf **(B)** (n = 4), 2 wpf **(C)** (n = 5) and 3 wpf **(D)** (n = 4). Note that no expression of *tbx4* is detectable in the lateral plate mesoderm at 18 hpf, 24 hpf, or 2 wpf, although it is detected in the pelvic fin bud as well as in the dorsal and anal fins at 3 wpf. af, anal fin; df, dorsal fin; pel, pelvic fin; sb, swim bladder. Scale bars, 0.5 mm.
